# Supplementary material for: Single-kernel near-infrared spectroscopy enables haploid kernel sorting in field and sweet corn using high-oil haploid inducers across diverse donor-inducer combinations
Source: Theor Appl Genet. 2026 Nov 13;139(12):332. doi: 10.1007/s00122-026-05424-1 (PMC13768295; doi:10.1007/s00122-026-05424-1)
Supplement: Supplementary file 1 [file 122_2026_5424_MOESM1_ESM.docx]

**Supplementary Material**

**Single-kernel near-infrared spectroscopy enables haploid kernel sorting in field and sweet corn using high-oil haploid inducers across diverse donor-inducer combinations**

Suman Sharma^1*^, Jeffery L. Gustin^2,3^, Ursula K. Frei^4^, A. Mark Settles^3,5^, Thomas Lübberstedt^4^, Márcio F. R. Resende Jr.^3^, Jenna Hershberger^1*^

^1^Plant and Environmental Sciences Department, Pee Dee Research and Education Center, Clemson University, Florence, SC, USA

^2^Maize Genetics Cooperation Stock Center, USDA-ARS, Urbana, IL, USA

^3^Horticultural Sciences Department, University of Florida, Gainesville, FL, USA

^4^Department of Agronomy, Iowa State University, Ames, IA, USA

^5^Space Biosciences Division, NASA Ames Research Center, Moffett Field, Mountain View, CA, 94035, USA

^*^Corresponding authors: [sumans@clemson.edu](mailto:sumans@clemson.edu), [jmhersh@clemson.edu](mailto:jmhersh@clemson.edu)

**Supplementary Tables**

**Supplementary Table 1** Two-way analysis of variance (ANOVA) estimation of the effect size (η²) of induction population donors and inducers on delta oil (Δ oil) and haploid induction rate (HIR) in 12 induction populations

| *Δ oil* | | | | | | |
| --- | --- | --- | --- | --- | --- | --- |
| **Source** | **SS** | **df** | **F** | ***p*-value** | **η²** | **η² (%)** |
| Donor | 1.6503 | 3 | 4.160 | 0.079 | 0.603 | 60.3% |
| Inducer | 0.4269 | 3 | 1.076 | 0.438 | 0.156 | 15.6% |
| Residual | 0.6611 | 5 | — | — | 0.241 | 24.1% |
| *HIR* | | | | | | |
| **Source** | **SS** | **df** | **F** | ***p*-value** | **η²** | **η² (%)** |
| Donor | 0.0044 | 3 | 7.604 | 0.026 | 0.540 | 54.0% |
| Inducer | 0.0028 | 3 | 4.814 | 0.062 | 0.342 | 34.2% |
| Residual | 0.0010 | 5 | — | — | 0.118 | 11.8% |

Type III SS. η² = SS_effect_/ SS_total_; df_error_ = 5

**Supplementary Table 2** Least squares (LS) means, 95% confidence interval (CI) and pairwise comparisons for donor and inducer effects on delta oil (Δ oil) and haploid induction rate (HIR)

| **Donor** | **Δ oil LS mean (%)** | **SE** | **95% CI** | **Group** | **HIR LS mean (%)** | **SE** | **95% CI** | **Group** |
| --- | --- | --- | --- | --- | --- | --- | --- | --- |
| CCO-2 | 1.28 | 0.182 | 0.59 – 1.97 | a | 9.25 | 0.70 | 6.60 – 11.90 | ab |
| Viking 60* | 1.83 | 0.426 | 0.21 – 3.46 | a | 8.88 | 1.63 | 2.65 – 15.10 | ab |
| Viking 51 | 1.88 | 0.182 | 1.19 – 2.57 | a | 10.50 | 0.70 | 7.85 – 13.15 | b |
| CCO-1 | 2.24 | 0.223 | 1.40 – 3.09 | a | 5.38 | 0.85 | 2.12 – 8.63 | a |

| **Inducer** | **Δ oil LS mean (%)** | **SE** | **95% CI** | **Group** | **HIR LS mean (%)** | **SE** | **95% CI** | **Group** |
| --- | --- | --- | --- | --- | --- | --- | --- | --- |
| IHO-2 | 1.58 | 0.223 | 0.73 – 2.42 | a | 9.63 | 0.85 | 6.37 – 12.88 | a |
| IHO-4 | 1.64 | 0.239 | 0.73 – 2.55 | a | 10.46 | 0.91 | 6.97 – 13.94 | a |
| IHO-3 | 1.98 | 0.239 | 1.07 – 2.89 | a | 7.13 | 0.91 | 3.64 – 10.61 | a |
| IHO-1 | 2.04 | 0.239 | 1.13 – 2.95 | a | 6.79 | 0.91 | 3.31 – 10.28 | a |

Least squares (LS) means estimated from main-effects model (Type III SS). Pairwise comparisons adjusted using Bonferroni correction (df error = 5). Means sharing the same letter are not significantly different at α = 0.05. *Viking 60 standard error (SE) is larger due to fewer combinations in the dataset.

**Supplementary Table 3** Classification error rates for 12 induction populations at the per-population optimal, lowest percentage (14%) and dynamic (offset = 2.0) thresholds for oil content-based classification

| **Population** | **HIR (%)^1^** | **Per-population Optimal** | | **Lowest Percentage (14%)** | | **Dynamic Threshold (offset = 2.0)** | |
| --- | --- | --- | --- | --- | --- | --- | --- |
|  |  | **FDR^2^** | **FNR^3^** | **FDR^2^** | **FNR^3^** | **FDR^2^** | **FNR^3^** |
| Viking 51 × IHO-1 (1)* | 8 | **0.19** | **0.00** | **0.20** | **0.36** | 0.21 | 0.53 |
| Viking 51 × IHO-2 (2) | 13 | 0.31 | 0.49 | 0.38 | 0.45 | 0.10 | 0.91 |
| Viking 51 × IHO-3 (3)* | 10 | **0.23** | **0.01** | **0.25** | **0.03** | **0.10** | **0.46** |
| Viking 51 × IHO-4 (4)* | 11 | **0.09** | **0.46** | 0.14 | 0.67 | 0.00 | 0.99 |
| Viking 60 × IHO-2 (5) | 10 | 0.38 | 0.42 | 0.40 | 0.60 | 0.43 | 0.91 |
| CCO-1 × IHO-1 (6) | 4 | 0.33 | 0.40 | 0.61 | 0.06 | 0.39 | 0.46 |
| CCO-1 × IHO-3 (7)* | 3 | **0.00** | **0.12** | 0.79 | 0.11 | **0.00** | **0.19** |
| CCO-1 × IHO-4 (8)* | 8 | **0.25** | **0.28** | 0.38 | 0.19 | 0.38 | 0.62 |
| CCO-2 × IHO-1 (9) | 8 | 0.64 | 0.40 | 0.71 | 0.55 | 0.00 | 0.97 |
| CCO-2 × IHO-2 (10) | 9 | 0.55 | 0.44 | 0.65 | 0.59 | 0.60 | 0.85 |
| CCO-2 × IHO-3 (11) | 8 | 0.64 | 0.43 | 0.70 | 0.56 | 0.79 | 0.95 |
| CCO-2 × IHO-4 (12) | 12 | 0.34 | 0.47 | 0.39 | 0.46 | 0.00 | 0.87 |
| **Mean** |  | **0.33** | **0.33** | **0.47** | **0.39** | **0.25** | **0.73** |

*Population met FDR ≤ 0.25 and FNR ≤ 0.50 operational benchmarks under the per-population optimal threshold. Bolded values indicate populations meeting the operational benchmarks

^1^Haploid induction rate

^2^False discovery rate

^3^False negative rate

**Supplementary Table 4** General model defective kernel classification error rates for three multivariate methods across 12 induction populations

| **Population** | **PLS-DA**‡ | | **SVM**‡ | | **RF**‡ | |
| --- | --- | --- | --- | --- | --- | --- |
|  | **FDR^1^** | **FNR^2^** | **FDR^1^** | **FNR^2^** | **FDR^1^** | **FNR^2^** |
| Viking 51 × IHO-1 (1) | 0.067 | 0.074 | 0.033 | 0.074 | 0.033 | 0.074 |
| Viking 51 × IHO-2 (2) | 0.026 | 0.000 | 0.019 | 0.000 | 0.061 | 0.000 |
| Viking 51 × IHO-3 (3) | 0.000 | 0.000 | 0.000 | 0.000 | 0.000 | 0.000 |
| Viking 51 × IHO-4 (4) | 0.030 | 0.000 | 0.020 | 0.008 | 0.035 | 0.019 |
| Viking 60 × IHO-2 (5) | 0.000 | 0.035 | 0.007 | 0.205 | 0.000 | 0.113 |
| CCO-1 × IHO-1 (6) | 0.046 | 0.000 | 0.034 | 0.000 | 0.038 | 0.170 |
| CCO-1 × IHO-3 (7) | 0.000 | 0.000 | 0.000 | 0.000 | 0.000 | 0.000 |
| CCO-1 × IHO-4 (8) | 0.000 | 0.000 | 0.000 | 0.171 | 0.063 | 0.141 |
| CCO-2 × IHO-1 (9) | 0.000 | 0.000 | 0.000 | 0.030 | 0.000 | 0.003 |
| CCO-2 × IHO-2 (10) | 0.000 | 0.000 | 0.000 | 0.051 | 0.000 | 0.067 |
| CCO-2 × IHO-3 (11) | 0.000 | 0.000 | 0.000 | 0.000 | 0.000 | 0.084 |
| CCO-2 × IHO-4 (12) | 0.000 | 0.000 | 0.004 | 0.000 | 0.062 | 0.000 |
| **Mean** | **0.014** | **0.009** | **0.010** | **0.045** | **0.024** | **0.056** |

‡Three multivariate methods: partial least squares discriminant analysis (PLS-DA), support vector machine (SVM), and random forest (RF)

^1^False discovery rate

^2^False negative rate

**Supplementary Table 5** Mean end-to-end performance metrics for the two-stage haploid kernel sorting pipeline across 12 induction populations

| **Population** | **Defective Fraction^1^** | **End-to-End Purity^2^** | **End-to-End Recovery^3^** |
| --- | --- | --- | --- |
| Viking 51 × IHO-1 (1) | 0.40 | 0.86 | 0.89 |
| Viking 51 × IHO-2 (2) | 0.12 | 0.80 | 0.61 |
| Viking 51 × IHO-3 (3)* | 0.00 | 0.98 | 0.56 |
| Viking 51 × IHO-4 (4) | 0.26 | 0.55 | 0.79 |
| Viking 60 × IHO-2 (5) | 0.09 | 0.53 | 0.86 |
| CCO-1 × IHO-1 (6) | 0.25 | 0.48 | 0.63 |
| CCO-1 × IHO-3 (7) | 0.32 | 1.00 | 0.79 |
| CCO-1 × IHO-4 (8) | 0.28 | 0.89 | 0.33 |
| CCO-2 × IHO-1 (9)* | 0.00 | 0.36 | 0.33 |
| CCO-2 × IHO-2 (10) | 0.06 | 0.23 | 0.70 |
| CCO-2 × IHO-3 (11) | 0.12 | 0.45 | 0.60 |
| CCO-2 × IHO-4 (12) | 0.31 | 0.98 | 0.31 |
| **Mean** | **0.18** | **0.68** | **0.62** |

*Populations ‘3’ and ‘9’ contained no defective kernels

^1^Defective fraction is the proportion of kernels classified as haploid by the initial haploid sorting stage that are actually defective kernels

^2^End-to-end haploid purity is the proportion of kernels classified as haploid by the complete two-stage pipeline that are truly haploid

^3^End-to-end haploid recovery is the proportion of actual haploid kernels correctly classified as haploid by the complete two-stage pipeline

**Supplementary Figures**

**Supplementary Fig. 1** Mean absorbance of the raw near-infrared (NIR) spectra of haploid, hybrid and defective kernels for each induction population [Viking 51 × IHO-1 (**1**), Viking 51 × IHO-2 (**2**), Viking 51 × IHO-3 (**3**), Viking 51 × IHO-4 (**4**), Viking 60 × IHO-2 (**5**), CCO-1 × IHO-1 (**6**), CCO-1 × IHO-3 (**7**), CCO-1 × IHO-4 (**8**), CCO-2 × IHO-2 (**9**), CCO-2 × IHO-2 (**10**), CCO-2 × IHO-3 (**11**), CCO-2 × IHO-4 (**12**)]. Absorbance is reported as log(1/Reflectance) for the NIR wavelength range of 940 to 1640 nm using the single-kernel NIR reflectance spectroscopy sorter. Populations ‘3’ and ‘9’ contained no defective kernels.

**Supplementary Fig. 2** Comparison of single-kernel near-infrared reflectance spectroscopy (skNIR)-predicted oil content (%) with nuclear magnetic resonance (NMR)-measured oil content (%) of the three kernel classes across 9 out of 12 induction populations.

**Supplementary Fig. 3** End-to-end 3 × 3 confusion matrices for haploid, hybrid, and defective kernel classification after the two-stage haploid kernel sorting pipeline across 12 induction populations. Rows represent the actual kernel classes, and columns represent the final predicted class. Correct classifications of the three kernel classes are shown in green, whereas misclassifications are shown in different colors according to the type of classification error: red indicates haploid contamination by hybrids and defectives, yellow indicates loss of haploids to the other two classes, and gray indicates other misclassifications between hybrid and defective classes. Values within each cell represent the average kernel counts across 20 replications in each actual-predicted category.
